# Major haplotype divergence including multiple germin-like protein genes, at the wheat *Sr2* adult plant stem rust resistance locus

**DOI:** 10.1186/s12870-014-0379-z

**Published:** 2014-12-30

**Authors:** Rohit Mago, Linda Tabe, Sonia Vautrin, Hana Šimková, Marie Kubaláková, Narayana Upadhyaya, Hélène Berges, Xiuying Kong, James Breen, Jaroslav Doležel, Rudi Appels, Jeffrey G Ellis, Wolfgang Spielmeyer

**Affiliations:** CSIRO Agriculture Flagship, Canberra, ACT 2601 Australia; INRA – CNRGV, 24 Chemin de Borde Rouge, Auzeville, CS 52627, 31326 Castanet Tolosan Cedex, France; Institute of Experimental Botany, Centre of the Region Haná for Biotechnological and Agricultural Research, Šlechtitelů 31, CZ-78371 Olomouc, Czech Republic; Key Laboratory of Crop Germplasm Resources and Utilization, MOA/Institute of Crop Sciences, CAAS/The Key Facility for Crop Gene Resources and Genetic Improvement, Beijing, 100081 PR China; Centre for Comparative Genomics, Murdoch University, Murdoch, 6150 WA Australia; Current address: Australian Centre for Ancient DNA (ACAD), University of Adelaide, Adelaide, SA 5005 Australia

**Keywords:** Adult plant resistance (APR), Map-based cloning, *Sr2*, Germin-like proteins (GLPs), Wheat stem rust, *Puccinia graminis*, Physical mapping, Gene expression

## Abstract

**Background:**

The adult plant stem rust resistance gene *Sr2* was introgressed into hexaploid wheat cultivar (cv) Marquis from tetraploid emmer wheat cv Yaroslav, to generate stem rust resistant cv Hope in the 1920s. Subsequently, *Sr2* has been widely deployed and has provided durable partial resistance to all known races of *Puccinia graminis* f. sp. *tritici*. This report describes the physical map of the *Sr2*-carrying region on the short arm of chromosome 3B of cv Hope and compares the Hope haplotype with non-*Sr2* wheat cv Chinese Spring.

**Results:**

*Sr2* was located to a region of 867 kb on chromosome 3B in Hope, which corresponded to a region of 567 kb in Chinese Spring. The Hope *Sr2* region carried 34 putative genes but only 17 were annotated in the comparable region of Chinese Spring. The two haplotypes differed by extensive DNA sequence polymorphisms between flanking markers as well as by a major insertion/deletion event including ten Germin-Like Protein (GLP) genes in Hope that were absent in Chinese Spring. Haplotype analysis of a limited number of wheat genotypes of interest showed that all wheat genotypes carrying *Sr2* possessed the GLP cluster; while, of those lacking *Sr2*, some, including Marquis, possessed the cluster, while some lacked it. Thus, this region represents a common presence-absence polymorphism in wheat, with presence of the cluster not correlated with presence of *Sr2*. Comparison of Hope and Marquis GLP genes on 3BS found no polymorphisms in the coding regions of the ten genes but several SNPs in the shared promoter of one divergently transcribed GLP gene pair and a single SNP downstream of the transcribed region of a second GLP.

**Conclusion:**

Physical mapping and sequence comparison showed major haplotype divergence at the *Sr2* locus between Hope and Chinese Spring. Candidate genes within the *Sr2* region of Hope are being evaluated for the ability to confer stem rust resistance. Based on the detailed mapping and sequencing of the locus, we predict that *Sr2* does not belong to the NB-LRR gene family and is not related to previously cloned, race non-specific rust resistance genes *Lr34* and *Yr36*.

**Electronic supplementary material:**

The online version of this article (doi:10.1186/s12870-014-0379-z) contains supplementary material, which is available to authorized users.

## Background

Wheat stem rust caused by the fungal pathogen *Puccinia graminis* Pers. f. sp*. tritici* (Pgt) is a major worldwide threat to wheat crops. The re-appearance of stem rust epidemics in East Africa and their spread eastward to Yemen and Iran, based on Ug99- a wheat stem rust isolate virulent on a large proportion of the world’s current wheat varieties, has alarmed the global wheat community [1,2]. More recently, new epidemics of stem rust caused by a different race of Pgt have been reported in Germany and Ethiopia (2013–2014; http://rusttracker.cimmyt.org/?page_id=40) reiterating the need to identify new resistance genes against rust pathogens, and to understand the mechanism of resistance, which may lead to development of novel strategies for crop protection.

Highly effective stem rust resistance was introgressed into hexaploid wheat cv, Marquis from the rust-resistant, cultivated emmer wheat cv Yaroslav from crosses initiated in 1916 by American breeder E.S. McFadden. Crosses were made between Yaroslav emmer and several varieties of hard red spring wheat, but were only successful with cv Marquis. Several years of selection for stem rust resistance, quality and yield among progeny derived from a single F_1_ plant resulted in the selections H_44–24_, H_29–24_ and H_35–24_. The line H_29–24_ was later released as the variety Hope [3]. High levels of field resistance against Pgt were observed in Hope and H_44–24_ (later re-named H44). This source of stem rust resistance was then used widely in the US, Australian and CIMMYT wheat breeding programs. McIntosh et al. [4] and Knott [5] genetically characterised the basis of resistance in Hope and H44, and identified three race-specific genes; *Sr7b* from the Marquis parent, and *Sr9d* and *Sr17* from Yaroslav. In addition Hare and McIntosh [6] described an adult plant, partial resistance gene, *Sr2*, effective against all tested Pgt strains. Using substitution lines carrying each Hope chromosome in Chinese Spring, Hare and McIntosh [6] mapped *Sr2* to chromosome arm 3BS. Selection in breeding programs for high levels of field resistance to stem rust derived from cv Hope combined *Sr2* with various seedling resistance genes, which resulted in effective stem rust resistance present in many wheat cultivars [7-10]. The partial resistance of *Sr2* has remained effective for almost a century in all wheat growing areas of the world, and is also effective against Ug99 and its derivatives. However, to be economically effective, *Sr2* must be combined with seedling resistance genes [11]. Consequently, the *Sr2* gene will continue to form an important component of resistance gene combinations required to control stem rust epidemics.

The resistance conferred by *Sr2* is characterized by a non-hypersensitive, partial resistance response, with varying effectiveness under field conditions. *Sr2* is one of the few race non-specific, adult plant, resistance (APR) genes currently used to provide protection against stem rust in wheat. *Sr2* stem rust resistance displays a dosage dependent resistance phenotype ("partial hemizygous effective" [6]), and is linked to pseudo-black chaff (PBC), a phenotypic trait that causes varying degrees of dark pigmentation on the stem internodes and glumes [8]. In certain backgrounds, *Sr2* is also associated with race specific seedling resistance to leaf rust caused by *P. triticina*, due to the action of two complementary resistance genes, *Lr27*, which is linked to *Sr2* [12] and the *Lr31* gene on chromosome 4B [8,13]. The basis of this complementary effect is unknown. We have demonstrated that a gene for resistance to wheat powdery mildew, *Blumeria graminis* f. sp. *triticae* (Bgt), also maps to the *Sr2* locus, but whether this resistance is race specific is unknown at this stage.

As a first step towards positional cloning of the gene, we previously mapped *Sr2* on the short arm of chromosome 3B, constructed a high resolution map of the region [14], and were unable to break the linkage between *Sr2, Lr27*, powdery mildew resistance and the phenotypic marker, PBC [12]. In the present paper, we produce a physical map of the *Sr2* region from Hope 3B, determine its DNA sequence and compare the haplotypes and gene content of the *Sr2* locus in Hope and Chinese Spring wheats. This analysis has revealed a major presence-absence polymorphism in tetraploid and hexaploid wheat for a cluster of ten germin-like protein genes.

## Methods

### Plant populations and phenotyping

The generation of the high resolution mapping family between Chinese Spring and the chromosome 3B substitution line Chinese Spring (Hope3B) and the stem and leaf rust rust phenotyping has been described previously [12,14]. Seeds of emmer (*Triticum turgidum* var. *dicoccum*) accessions including cv Yaroslav and of *Triticum aestivum* cv Marquis and cv Hope were obtained from the Australian Winter Cereal Collection, Tamworth. The Chinese Spring 3BS deletion stock (3BS-8) was obtained from Wheat Genetic and Genomic Resource Center, Kansas State University, USA. The CAP lines were part of the US Wheat Coordinated Agricultural Project (CAP) and kindly provided by Gina Brown-Guedira USDA.

### Construction of the 3B-specific BAC library from cv. Hope

The BAC library from chromosome 3B of cv. Hope consisted of two sub-libraries designated TaaHop3BFhA and TaaHop3BFhB, respectively. Both sub-libraries were prepared from the DNA of chromosome 3B purified by flow-cytometric sorting [15,16] (Additional file 1: Figure S1). Purified genomic DNA was partially digested with *Hin*dIII and cloned into the pIndigoBAC-5 vector (Epicentre, Madison, USA). The TaaHop3BFhA sub library was constructed from a limited number of chromosomes (10^6^, corresponding to ~1.8 μg DNA) as described in Šafář et al. [17], with some modifications. The smaller amount of DNA was compensated by lower than normal stringency size selection, including just one size-selection step and usage of DNA fractions of a relatively smaller size (55–80 kb and 80–115 kb). This resulted in a BAC library consisting of 92,160 clones, with an average insert size of 78 kb. This sub-library represented ~6-fold coverage of chromosome 3B. The TaaHop3BFhB sub library was constructed from 2 × 10^6^ chromosomes (~3.6 μg DNA) by applying two size-selection steps as described in Šimková et al. [18]. The higher amount of DNA allowed efficient cloning of larger DNA fragments (145–220 kb), resulting in a library of 43,776 BAC clones with mean insert size of 160 kb, which also represented ~6-fold coverage of chromosome 3B. Screening of the library was done using ^32^P labeled DNA probe using standard protocols.

### Construction of a non-gridded BAC library from cv. Hope

A second BAC library designated Tae-B-Hope-ng was constructed from cv Hope genomic DNA without chromosome sorting, at the Centre National de Resources Génomiques Végétales (CNRGV)- INRA, France. It was produced using a non-gridded BAC library construction protocol based on Isidore et al. [19] with the following modifications: (1) growth of pooled BAC clones in liquid LB medium; (2) BAC pools DNA amplification by whole genome Genomiphi.v2 phi29 enzyme kit (GE Healthcare) instead of DNA extraction; (3) use of secondary pooling steps in order to identify positive clone coordinates with less screening effort after clone picking. High molecular weight (HMW) DNA was prepared from 20 g of leaf material from cv. Hope. Embedded HMW DNA was partially digested with *Hind*III (Sigma-Aldrich, St-Louis, Missouri), size selected, eluted and ligated into pIndigoBAC-5 *Hind*III-Cloning Ready vector (Epicentre Biotechnologies, Madison, Wisconsin). Two successive HMW DNA extractions and three independent sizing steps led to production of 336,670 BAC clones (101 kb average insert size) representing a total ~2-fold coverage of genome. BAC clones were divided into 268 pools before overnight growth and DNA amplification. Pools were screened using specific PCR markers previously developed from clones in the 3B sorted library. The primer sequences for the probe PCR fragments and PCR markers are summarized in Additional file 2: Table S1.

### BAC sequencing and sequence assembly

BAC clones from the *Sr2* locus of Hope were isolated using probes from the previously characterised 3B locus of Chinese Spring and were sequenced by a BAC-by-BAC shotgun method at 10× coverage using Applied Biosystems capillary sequencing. Base calling, quality trimming and assembly of the raw sequence reads into contigs were done using the PHRED/PHRAP/CONSED package [20,21] and GAP4 [22] from the Staden package was used for data integration and sequence finishing. The BACs isolated from the non-enriched Hope genomic DNA library were sequenced using Roche 454 sequencing platform. A hybrid assembly of the sequence from 454 and Sanger sequencing was done using Newbler v. 2.3. Additional gap closure was performed on the assembled scaffold sequences by sequencing pooled BAC DNA and an 8 kb paired end library using Roche 454 sequencing platform. Scaffolds were also assembled using Sequencher v. 5.2. Annotated sequence of *Sr2* region in Hope has been deposited in GenBank (Acc no. KP244323).

### Gene annotation: gene prediction

Genes were annotated using the gene prediction software packages FGENESH (www.softberry.com), AUGUSTUS (http://bioinf.uni-greifswald.de/augustus/) and GENSCAN (http://genes.mit.edu/GENSCAN.html). Gene calls were also made using CLC Genomics workbench v, 7.04. The predicted gene sequences were used for BLASTX and BLASTP analysis against protein databases (NCBI protein and Swiss-prot databases) to identify candidate genes and determine whether they were full length. Intron-exon structure of predicted genes was refined by BLAST comparisons of the predicted gene sequences with the Expressed Sequence Tag (EST) database at NCBI (http://www.ncbi.nlm.nih.gov/dbEST/).

### Gene annotation: RNA-seq

Further expressed sequences in the *Sr2* region were identified by aligning RNA-seq reads from leaves of cv Hope with the *Sr2* genomic sequence. Because of the association of mildew and rust resistance at the *Sr2* locus, this RNA was isolated from plants that were inoculated with mildew at four weeks after sowing by applying spores of a glasshouse isolate of Bgt to the abaxial surface of the leaves with a dry paint brush, followed by application of a fine spray of water. Plants were maintained under a shade curtain in the glasshouse at 23°C, in tubs of shallow water to increase humidity, and leaves were sampled at 15 hours after inoculation, and at five days after inoculation. Total RNA was isolated using a Qiagen RNA-Easy Plant Mini-kit, and RNA was DNase-treated on the column using the Qiagen protocol. A pooled sample containing 50% each of DNase-treated RNA isolated from leaves collected at 15 hours and five days after inoculation was sequenced by the Australian Genome Research Facility. RNA-seq was quality trimmed and aligned to the Hope *Sr2* genomic sequence using CLC-Genomics Workbench. Expressed regions of the genomic sequence, for which RNA-seq reads aligned at 100% identity, were annotated by BLASTN comparison with DNA sequence databases.

### Mapping

Gene sequences identified in the *Sr2* region of Hope and CS were mapped as PCR markers (Additional file 2: Table S1) using the high resolution mapping family described previously [12,14]. The specificity of PCR markers to chromosome 3B was confirmed using the DNA of the Chinese Spring 3BS-8 deletion line. PCRs were performed in a volume of 20 μl with 10 mM of each primer, 0.2 mM dNTPs, 1.5 mM MgCl_2_, 1.0 units GoTaq DNA polymerase (Promega) and 100–200 ng of template DNA. The annealing temperature used for PCR was generally 3-4°C below the T_m_ of the primers used.

## Results

### A BAC contig for the *Sr2* region in the stem rust resistant cv Hope

**Figure 1:**
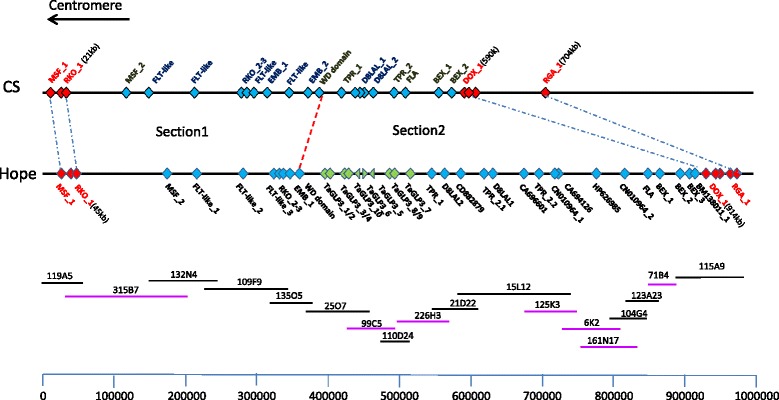
**Physical map of the** ***Sr2*** **region from CS showing position of annotated genes** **[** 24 **]** **and its comparison with corresponding region in Hope.** The recombining markers/genes RKO_1 and DOX_1 are 568 kb apart in CS as compared to 868 kb in Hope and are shown in red. A group of ten genes annotated as TaGLP3’s in Hope (position 399-510 kb) are indicated by green colour. Recombining genes are indicated in red. All other genes are shown in blue. The minimum tiling path consisting of 18 BAC clones from the *Sr2* physical map from cv Hope is shown at the bottom. The clones in black were isolated from the 3B Hope enriched library while clones in purple were isolated from the PCR screening of the pooled genomic BAC library from Hope.

Although the sequence information from the non-*Sr2* carrying wheat Chinese Spring (henceforth CS) wheat was useful initially [23,24], the characterisation of the locus required BAC clones from the *Sr2* carrying region of Hope wheat. A BAC library was prepared from flow sorted chromosome 3B of Hope and screened with low copy markers derived from the ends of CS BAC clones. A total of 11 BACs were identified that formed six contigs (Figure 1). Efforts to isolate additional BAC clones from this library, and to close the gaps between the contigs were unsuccessful. To complete the physical map, an additional whole genome BAC library was developed from Hope, and screened with markers flanking the gaps. The positions of the seven newly selected BAC clones were confirmed on the basis of sequence identity with overlapping regions from the BACs derived from the 3B chromosome-sorted library and also genetic mapping of markers which were derived from these new clones. In total, the 18 BAC clones formed a single contig of 968 kb, and included the distal and proximal markers *RKO_1* and *DOX_1* that recombined with *Sr2*. The same interval in CS was 567 kb long [24] indicating that the two haplotypes were substantially divergent and included deletion/insertion events. The DNA sequence of the 968 kb *Sr2* region (Genbank KP244323) was determined by sequencing all 18 BAC clones.

### Gene annotation of the Hope *Sr2* genomic region sequence and comparison to CS

Gene annotation of the 968 kb *Sr2* region was done using gene prediction software and by comparison with the published CS genomic sequence covering the region (Genbank accession FN645450). In addition, sequences from the Hope *Sr2* region that were expressed in pathogen challenged leaves of Hope were identified by alignment of RNA-seq data with the *Sr2* region DNA sequence. Table 1 lists sequentially all identified genes and their sequence coordinates in the *Sr2* genomic region of Hope compared to the corresponding region of the previously annotated CS sequence. The annotation identified 34 genes and gene fragments from the Hope *Sr2* cosegregating region between the *RKO_1* and *DOX_1* flanking markers whereas only 17 genes and gene fragments were identified in the corresponding region of CS.

**Table 1:** **Gene annotation of Hope** ***Sr2*** **region and comparison with the corresponding region in CS**

**No.**	**Name**	**Annotation**	**Hope gene start**	**Hope gene end**	**CS gene id**	**CS gene pos**
1	MSF_1^$^	Male sterility factor	29132	35097	ctg0011b.00010.1	8024
2	EP_1^$^	Hypothetical protein	39298	41832	ctg0011b.00020.1	16001
3	RKO_1^$^	Receptor kinase	45570	48036	ctg0011b.00030.1	21207
4	MSF_2^$^	Male sterility factor	174463	180047	ctg0011b.00040.1	130246
5	Flowering Locus T-like_1	Flowering Locus T-like protein	207129	208229	ctg0011b.00050.1	154860*
6	Flowering Locus T-like_2	Flowering Locus T-like protein	274176	275721	ctg0011b.00060.1	218850
7	Flowering Locus T-like_3	Flowering Locus T-like protein	322283	323263	ctg0011b.00070.1	281996
8	RKO_2	receptor kinase	329447	331621	ctg0011b.00080.1	293102
9	RKO_3	receptor kinase	332015	335105	ctg0011b.00090.1	295683
10	EMB_1	PPR repeat domain containing protein	339212	341395	ctg0011b.000100.1	307937
11	Flowering Locus T-like^#^	Flowering Locus T-like protein	-	-	ctg0011b.000110.1	351119
12	EMB_2^#^	PPR repeat domain containing protein	-	-	ctg0011b.000120.1	379263
13	WD domain*	similar to WD domain, G-beta repeat	349395	355055	ctg0011b.000130.1	385794
14	GLP_1	Germin-like protein	398118	398929		-
15	GLP_2	Germin-like protein	399614	400424		-
16	GLP_3	Germin-like protein	423764	424562		-
17	GLP_4	Germin-like protein	425172	425984		-
18	GLP_10*	Germin-like protein	444653	445216		
19	GLP_6*	Germin-like protein	455726	456520		-
20	GLP_5*	Germin-like protein	463024	463811		-
21	GLP_8	Germin-like protein	474211	475020		-
22	GLP_9	Germin-like protein	475777	476588		-
23	GLP_7	Germin-like protein	508822	509633		-
24	TPR1	Tetratricopeptide repeat domain containing protein	546645	549809	ctg0011b.000140.1	435705
25	D8LAL2	Hypothetical protein	562871	565156	ctg0011b.000160.1	447301
26	CD882879	Hypothetical protein	587682	589820		464713
27	TPR2*	Tetratricopeptide repeat domain containing protein	613921	615236	ctg0011b.000170.1	498370
28	D8LAL1	Hypothetical protein	625926	626996	ctg0011b.000150.1	443924
29	CA696601	Hypothetical protein	670987	671361		
30	TPR2*	Tetratricopeptide repeat domain containing protein	692673	695113	ctg0011b.000170.1	498370
31	CN010964	Hypothetical protein	710337	711484		574223*
32	CA694126	No annotation	712343	712774		507295*
33	HP626985	Hypothetical protein	776063	776392		507441*
34	CN010964	Hypothetical protein	809317	810476		574223*
35	FLA30	fasciclin-like arabinogalactan precursor	840622	841206	ctg0011b.000180.1	510269
36	BEX_1	beta-expansin 1a precursor	846394	847826	ctg0011b.000190.1	561427
37	BEX_2	beta-expansin 1a precursor	854813	856068	ctg0011b.000200.1	570188
38	BEX_3	beta-expansin 1a precursor	892334	893409		
39	BM138011_1	BTB-domain containing protein	909112	909465		
40	DOX_1^$^	disulfide oxidoreductase	914790	916571	ctg0011b.000210.1	590774
41	CA681101^$^	Hypothetical protein	923311	923709		599356
42	BM138011_2^$^	BTB-domain containing protein	926622	926983		602889
43	BTB-domain containing protein^$^		957404	959539		109661*
44	RGA_1^$^*	similar to disease resistance RPM1-like protein	960894	966749	ctg0011b.000220.1	704997

^$^Recombining genes; *partial genes/pseudogenes; Not present in Hope.

**Figure 2:**
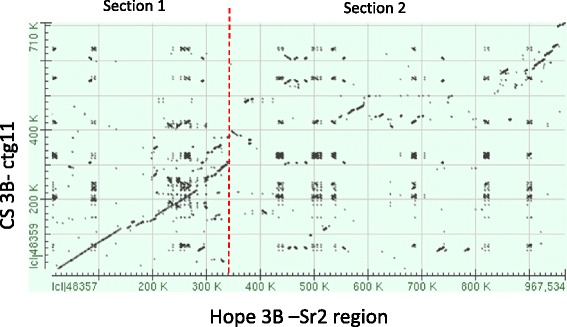
**Dot-matrix plot of the Hope (3B)** ***Sr2*** **region with the corresponding region on CS (3B; part of ctg11, GenBank FN645450).** Section 1 denotes region between 45–360 kb, section two includes 360-915 kb region.

Based on the level of sequence identity between CS and Hope, the *Sr2* locus was divided into two sections (Figures 1 and 2). The proximal section (section 1, 45 kb to 360 kb) of the Hope *Sr2* locus sequence showed high similarity with the corresponding region from CS. Eight genes were annotated in section 1 of both Hope and CS, with 2 additional genes present in CS (Table 1) [24]. The length and gene content of this sub-region were closely comparable between CS and Hope, except for a segment of approximately 40 kb in CS, containing a Flowering Locus T– like gene and EMB_2, which were missing in Hope (Table 1). Most of the shared genes in section 1 contained several DNA sequence polymorphisms between the CS and Hope, and their predicted protein products differed by one or more amino acid polymorphisms.

In section two (360 kb to 915 kb; Figures 1 and 2), distal to the shared WD-domain pseudogene, the Hope and CS regions differed markedly. In this region, 26 putative genes were annotated in Hope, whereas only seven genes or gene fragments were annotated in CS. (Table 1). Small regions of high similarity between Hope and CS were evident at 560-590 kb and at 900-968 kb (Figure 1) but apart from these islands of similarity, the two DNA sequences diverged radically. Most notably, an insertion of approximately 100 kb containing ten genes annotated as germin-like proteins (GLPs) was present in Hope, positioned between CS gene ids ctg0011b.000130.1 and 140.1 (Table 1). Despite the lack of overall sequence similarity across the distal part of the locus, other genes that were annotated between WD-domain and DOX_1 were present in both haplotypes (Table 1, Figure 1). This section of the *Sr2* locus contained several genes of unknown function (hypothetical proteins, Table 1) that were identified by alignment of the genomic sequence with RNA seq data. These expressed sequences were not annotated as putative genes in the published CS sequence (FN645450), and are listed with the accession number of the most similar GenBank EST in Table 1. For CA696601, CN010964, CA694126, HP626985, CN010964 and BM138011, the sequences in Hope were predicted to encode small proteins (e.g. 84 amino acids for CN010964), but in CS the putative genes were truncated. Overall, the DNA regions that encode these predicted proteins showed 80 to 92% DNA sequence identity between the *Sr2* haplotypes. CD882879 encoded a predicted protein of 374 amino acids in Hope and 378 amino acids in CS. Re-sequencing of the gene from CS revealed an error in the deposited FN645450 CS sequence, in which insertion of an extra nucleotide disrupted the open reading frame, resulting in the gene not being annotated in the original CS genomic sequence [24]. The protein coding regions of the CD882879 genes from Hope and CS shared 92% DNA identity, predicting proteins with 90% amino acid identity.

Hypothetical genes D8LAL1 and D8LAL2 were positioned 4 kb from each other in the CS genomic sequence. In contrast, the relative position of these genes was reversed and 60 kb apart in Hope, indicating that inversion and insertion events occurred at this position between the two haplotypes. Predicted genes encoding a fasciclin-like arabinogalactan precursor (FLA; gene id ctg0011b.000180.1), two beta-expansin 1a precursor proteins (BEX_1 and BEX_2; gene ids ctg0011b.000190.1, ctg0011b.000200.1), and a putative disulfide oxidoreductase (DOX_1; gene id ctg0011b.000210.1) formed the distal end of the *Sr2* locus in both Hope and CS. All these genes were also present in the corresponding Hope region, as well as an additional copy of the truncated tetratricopeptide repeat domain containing protein, TPR_2 and a beta-expansin precursor, annotated as BEX_3 (Table 1).

### Mapping *Sr2* with respect to newly derived DNA markers

To establish the region that co-segregated with *Sr2*, PCR markers developed from polymorphic regions of the Hope and CS sequences were mapped in the high resolution mapping family developed by Kota et al. [14]. At the proximal end of the *Sr2* region, the marker from the MSF_1 gene (CS gene id ctg0011b.00010.1) was separated from *Sr2* in two recombinants, while the marker from the RKO_1 gene (CS gene id ctg0011b.00030.1) was separated by one recombinant. At the distal end, marker RGA_1, derived from a nucleotide binding-leucine rich repeat gene (NB-LRR), was separated from *Sr2* in two recombinants, while DOX_1 (CS gene id ctg0011b.000210.1) was separated in a single recombinant. Markers developed from genes between RKO_1 and DOX_1 co-segregated with *Sr2*. The genes shared between Hope and CS all encoded proteins with one or more polymorphisms between the two haplotypes, and several genes, including the GLPs were specific to Hope. Thus, sequence comparisons alone were unable to pinpoint a specific candidate for *Sr2*.

### *Sr2*-linked GLPs

**Figure 3:**
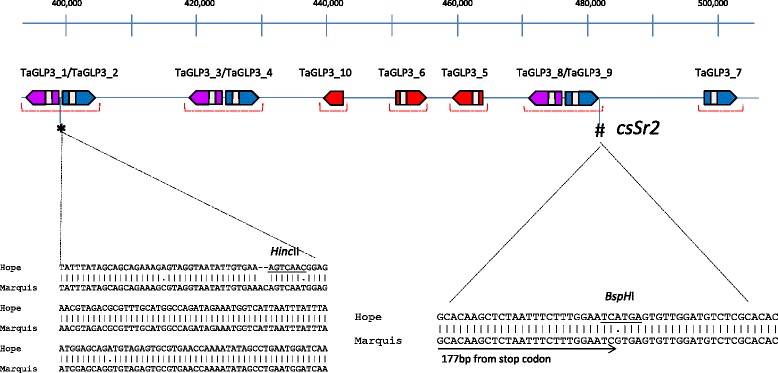
**Organization of TaGLP3 genes at the CS (Hope3B)** ***Sr2*** **locus.** GLPs belonging to same group are shown in the same color (blue or purple). The pseudo genes are shown in red. The red bracket underneath the GLPs indicates the region PCR amplified from Marquis and sequenced. The asterisk (*) shows the position of the SNPs/ indels between Hope and Marquis between TaGLP3_1/2 and the hash symbol (**#**) shows the position of CAPs marker csSr2 [27]. The sequence comparison between Hope and Marquis showing the respective SNPs/indels is shown at the bottom.

**Figure 4:**
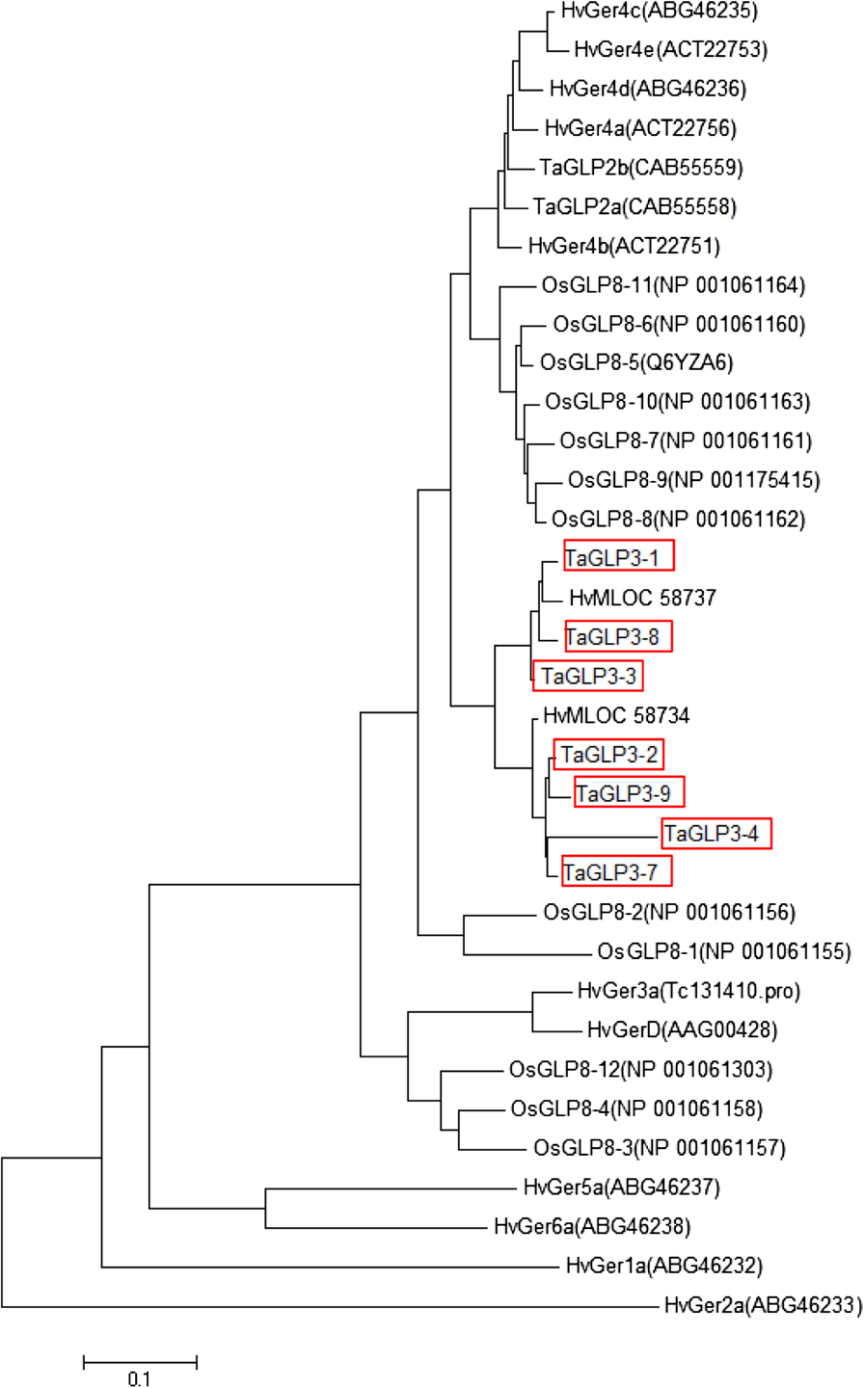
**Phylogenetic tree of predicted amino acid sequences of full length TaGLP3 genes from the Hope** ***Sr2*** **locus compared with the most similar available sequences from barley, rice and wheat, most of which were reported to be involved in disease resistance** **[** 25 **,** 26 **,** 36 **,** 37 **].** The phylogenetic tree was constructed by neighbour-joining method using MEGA program version 6.0. TaGLP3s are indicated by a red border.

The GLP genes co-segregating with *Sr2* were of particular interest because of the reported association of GLPs with plant defence ([25] and references therein). Following the convention used for naming GLPs on chromosome 8 of rice [26], the ten GLPs from the *Sr2* locus were named TaGLP3_1 to TaGLP3_10 (*Triticum aestivum* Germin-like proteins from chromosome 3, numbered from proximal to distal). TaGLP3_5, 6 and 10 were pseudogenes because they encoded truncated proteins compared to the other GLPs. Of the remaining seven apparently full-length genes, six, (TaGLP3_ 1/2, −3/4 and - 8/9) were organised as divergently-transcribed pairs, each separated by 610–675 bps between predicted ATG translation start codons, presumably constituting a short, shared promoter (Figure 3). Only TaGLP3_7 was present as a single gene. Sequence comparison showed that the *Sr2* locus TaGLP3s could be organised into 2 sequence groups (Table 2 and Figure 4). One group consisted of GLPs 1, 3, 6, 8 and 10 (TaGLP3_1 group), while GLPs 2, 4, 5, 7 and 9 (TaGLP3_2 group) formed the second category. The sequence identity between members within a group ranged between 93-98% while the identity between the GLPs in different groups was 77-82%. For divergently transcribed genes, the gene pair consists of one member of each group. Comparisons of the derived amino acid sequences of the wheat TaGLP3s with sequences from wheat, barley and rice showed that the TaGLP3s were most closely related to a previously undescribed, single, divergently transcribed pair of barley GLPs at a putatively orthologous locus on chromosome 3H (http://plants.ensembl.org/Hordeum_vulgare/Info/Index). Less similar sequences were also present in other cereals, including rice and Brachypodium, although not necessarily at syntenic genomic locations. The TaGLP3s and the orthologous barley GLP gene pair products shared more than 90% amino acid identity with each other, and shared 80 to 84% identity with the next most similar sequences in barley, the Ger4 sub-family, and with the most similar sequences in rice, a group of Ger4 sub-family OsGLP8s on chromosome 8 (Figure 4).

**Table 2:** **Comparison of Hope** ***Sr2*** **locus TaGLP3 predicted protein sequences**

		**1**	**2**	**3**	**4**	**5**	**6**	**7**
1.	TaGLP3_1	100						
2.	TaGLP3_2	86	100					
3.	TaGLP3_3	97	88	100				
4.	TaGLP3_4	78	89	79	100			
5.	TaGLP3_7	85	96	87	88	100		
6.	TaGLP3_8	96	86	96	77	85	100	
7.	TaGLP3_9	85	96	87	87	97	85	100

### Amplification of TaGLP3s from hexaploid and tetraploid wheat

To further investigate the TaGLP3 presence/absence polymorphism in other wheat genotypes, we designed PCR primers based on Hope coding and non-coding sequences for all the TaGLP3s (including approximately 1 kb flanking sequence) to determine if the large insertion carrying these GLPs was present in other wheats. All GLPs amplified from genomic DNA of the wheat cultivar Marquis (see Figure 3 for regions which were sequenced in Marquis), which was the original recipient of *Sr2* from emmer, while none could be amplified from CS indicating that this polymorphism existed in hexaploid wheat before introgression of *Sr2*. Three SNPs and two nucleotide insertions were identified in the shared promoter of TaGLP3_ 1/2 between Hope and Marquis (Figure 3). Sequencing of the promoter region and predicted coding sequences of TaGLP3_1 and 3–2 from diverse germplasm including CAP10, CAP12, CAP13, CAP16, CAP30, CAP31 and CAP32 revealed only the Hope or Marquis haplotypes (Table 3). One of these polymorphisms resulted in loss of a *Hinc*II restriction site in the Marquis sequence and was used to develop a CAPS marker (GLP-1/2 CAPs). A second SNP occurred 3’ of TaGLP3 -9 and was previously developed as a diagnostic PCR marker for *Sr2*, *csSr2* [27]. PCR was used to assess the presence of the TaGLP3s in a range of hexaploid and tetraploid wheat genotypes (Table 3). Based on the PCR screening, four haplotypes were identified: 1. Both PCR products did not amplify (null), 2. Both PCR products carried the Marquis alleles (MM), 3. Both PCR products carried the Hope alleles (HH), 4. TaGLP3_1/2 CAPs generated the Hope allele, while *csSr2* amplified the Marquis allele (HM). The tetraploid durums and nine out of the 11 emmers were null for both markers. Yaroslav and AUS 3511 emmer contained the Hope allele for both markers (HH), while a second emmer (AUS19385) carried the Hope allele at TaGLP3_ 1/2 and the Marquis allele for *csSr2* (HM). In hexaploid wheat, all four haplotypes were present (Table 3). The csSr2 marker was diagnostic for the presence of *Sr2* in a wide range of wheat genotypes [27], however the TaGLP3_1/2 CAPs marker detected the Hope allele in several wheats that lacked *Sr2* confirming that *csSr2* is the more useful marker (Table 3). Most significantly, no differences were detected between Hope and Marquis in the coding regions of the GLP genes.

**Table 3:** **Marker survey of TaGLP3 CAPs markers in tetraploid and hexaploid wheats**

**Genotype**	***Sr2***	**TaGLP3_1/2 CAPs**	**csSr2**
Langdon	-	-	-
Bansi	-	-	-
Glossy Huguenot	-	-	-
Emmer (AUS 3511)	?	H	H
Emmer (AUS 3728)	?	-	-
Emmer (AUS 3741)	?	-	-
Emmer (AUS 3743)	?	-	-
Emmer (AUS 3745)	?	-	-
Emmer (AUS 3748)	?	-	-
Emmer (AUS 10731)	?	-	-
Emmer (AUS 11436)	?	-	-
Emmer (AUS 15520)	?	-	-
Emmer (AUS 18175)	?	-	-
Emmer (AUS 19385)	?	H	M
Yaroslav Emmer (AUS 2789)	+	H	H
Chinese Spring	-	-	-
CS/Hope(3B)	+	H	H
Marquis	-	M	M
Thatcher	-	M	M
CAP10 [Q36 (OR9900553)]	-	H	M
CAP12 (Penawawa)	?	H	H
CAP13 (Finch)	-	H	M
CAP16 (NY18/CC 40–1)	?	M	M
CAP30 (2174)	?	H	M
CAP31 (Weebill 1)	?	H	M
CAP32 (Jupateco 73S)	?	H	M
Aroona	-	-	-
Arrino	-	-	-
Turmbull	-	H	M
Brookton	-	-	-
Cadenza		-	-
Diamondbird	+	H	H
Dollarbird	+	H	H
Federation	-	-	-
Pavon	+	H	H
Sunstate	+	H	H
Purplestraw	-	H	M
Fultz	-	H	M

Note: H = Hope type, M = Marquis type, − = Null,? = not known.

## Discussion

### *Sr2* is not related to NB-LRR resistance genes or APR genes *Lr34* and *Yr36*

In this study we have determined the sequence of a region of the wheat chromosome carrying the *Sr2* adult plant stem rust resistance gene from cv Hope, and compared DNA sequence and gene content with the non-*Sr2* cultivar Chinese Spring that was used as the rust susceptible parent in a high resolution mapping of *Sr2* [12,14]. There were no nucleotide binding site leucine rich-repeat (NB-LRR) genes, the most frequent class of resistance gene, that co-segregated with *Sr2*, which indicates that *Sr2* is not a member of this class. Further, no sequences related to other cloned APR genes such as *Lr34* or *Yr36* occured in the region [28,29]. There were many polymorphisms, including large indels between the two haplotypes, preventing identification of *Sr2* from sequence data alone.

### Haplotype divergence and gene cloning

The *Sr2* region of Hope and CS diverged in gene content, including the presence in Hope, and absence in CS, of a cluster of ten GLP genes. Large insertion-deletion events have been reported at other loci in wheat and in other cereals. For example, physical mapping of the adult plant stripe rust resistance locus *Yr36* [29] showed a 183 kb insertion in the resistant line RSL65 compared to the susceptible durum wheat Langdon. This region, carrying four genes including the *Yr36* resistance gene, originated from the wild species *T. turgidum* var. *dicoccoides*. Similarly, a deletion of 351 kb was reported in *T. turgidum* var. *dicoccoides* compared to Langdon in the region containing *Tsn1*, a gene that confers sensitivity to the toxin ToxA in tan spot disease of wheat [30,31] In rice, large sequence variation between a wild donor species and a recurrent parent was reported for the yield-improving QTL, q-GY2 [32]. In this study, we report major haplotype divergence within the hexaploid wheat species, indicating that large insertion/deletion events may be common, and not restricted to comparisons between inter-species haplotypes. Such major differences will impede rapid, map-based identification of target genes in specific wheat genotypes, even when high quality, reference genome sequence of CS becomes available. It is also likely that haplotype divergence, especially the large insertion/deletion of GLPs in the *Sr2* region, resulted in the suppression of recombination and lack of genetic resolution of the target region. Consequently, a new mapping family was established by using Marquis, which carries the GLP gene cluster, as the susceptible parent. Initial results have indicated that in this family, recombination occurred within the region that previously co-segregated with *Sr2*. The characterisation of new recombinants is in progress.

### What is the possible evolutionary value of the presence /absence GLP polymorphism on 3B?

It has been suggested that GLP functional diversity has arisen via sequential gene duplication events, followed by mutation, including in flanking regulatory regions, giving rise to diversity in temporal and spatial gene expression patterns [25]. Diverse wheats reflect four GLP haplotypes in the *Sr2* region; *Sr2* types with the Hope allele at TaGLP3_9 and TaGLP3_1/2, Marquis types with the Marquis allele at TaGLP3_9 and either the Marquis or Hope allele at TaGLP3_1/2, and CS types with no GLPs at the locus (Tables 2 and 3) [27]. The Hope haplotype with *Sr2* and stem rust resistance has been selected by breeders. It is possible that there is an as yet undefined selection acting for or against the Marquis (GLP +) and CS (GLP -) haplotypes in different geographic regions, which may account for the wide spread occurrence of the chromosome 3B presence/absence polymorphism in *Sr2-* wheat genotypes. This is reminiscent of the presence/absence polymorphism for the resistance gene RPM1 in *Arabidopsis thaliana* field populations that has been interpreted to result from balancing selection [33].

### Germin-like proteins

The GLP genes at the *Sr2* locus are of interest because of the reported association of this category of genes with plant defence responses. GLPs are small (~220 amino acid residues), secreted, functionally and taxonomically diverse cupin domain-containing proteins, two of which are reported to produce hydrogen peroxide, a plant defence signal, while several other members of the protein family have been associated with other enzymatic functions [34]. Sequence analysis showed that TaGLP3s at the *Sr2* locus were most similar to a pair of divergently-transcribed GLPs at a syntenic position on barley chromosome 3H (Figure 4). The TaGLP3s and this orthologous barley locus, which is distinct from GerD previously mapped on barley chromosome 3H [35], constitute a novel sub-family of GLPs which has not been studied previously, and as far as we are aware, the only sub-family transcribed divergently from short intergenic promoters. The next closest GLP sub-family relative of the *Sr2* locus sub-family is the GER4 class of GLP proteins in wheat, barley and rice, members of which have all been functionally associated with fungal disease resistance (Figure 4) ([25] and references therein). Transient over-expression of HvGER4 or HvGER5, as well as transient silencing by RNA interference of HvGER3 or HvGER5 [36] protected barley epidermal cells from attack by powdery mildew. Transient expression of wheat orthologs of HvGER4, germin-gf-2.8, TaGLP2a and TaGLP2b (which have identity between 72-82% with TaGLP3s) in epidermal cells also reduced the penetration efficiency of the powdery mildew fungus *Blumeria graminis* f. sp. *hordei* in wheat [37]. No experiments involving over-expression of GLPs in stable transgenic wheat or barley that confirm a direct role of these genes in disease resistance have been reported. Manosalva et al. [26] identified a QTL for rice blast resistance containing a cluster of 12 GLPs on rice chromosome 8, consisting of both GER3 and GER4 sub-family GLPs. RNA interference studies in transgenic rice showed that as more OsGLP genes were suppressed, disease susceptibility of the plants increased. Of the 12 OsGLPs, the OsGLP8-4 member (ortholog of HvGER4) was most effective in providing resistance. The resistance conferred by this QTL was effective against the rice blast and sheath blight pathogens. Despite the sequence similarity between the TaGLP3 gene cluster at the *Sr2* locus and GLPs from other cereals that are associated with disease resistance, our data show that the predicted amino acid sequences of the TaGLP3s are identical between the stem rust sensitive cv Marquis, and the resistant cv Hope. Future analysis will determine whether the sequence polymorphisms adjacent to TaGLP3_1,2 or 9 are associated with any differences in gene expression between Hope and Marquis.

## Conclusions

In this study, we completed the physical map of the *Sr2* locus in stem rust resistant cv Hope. Sequence comparison with susceptible CS revealed major haplotype divergence including deletion/insertion events carrying a large cluster of GLPs in Hope. The GLPs were however present in Marquis, a stem rust susceptible wheat not carrying *Sr2*. Haplotype divergence within hexaploid wheat may prevent rapid map-based identification of target genes in specific wheat genotypes even when high quality, reference genome sequence is available. Candidate genes within the *Sr2* region are being evaluated for their capacity to confer stem rust resistance.

## Additional files

Additional file 1: Figure S1. Histogram of relative fluorescence (flow karyotype) from flow cytometric analysis of DAPI-stained mitotic chromosomes of *Triticum aestivum* cv. Hope. The flow karyotype consists of three composite peaks I – III representing groups of wheat chromosomes and peak of chromosome 3B. Inset: Images of flow-sorted 3B after FISH with a probe for GAA microsatellite (green). The chromosomes were counterstained by DAPI (blue). X axis: Relative fluorescence intensity. Y axis: Number of particles. Additional file 2: Table S1. Primer sequences used for amplification of markers that were genetically mapped.
