# Supplementary figures and images for: Major haplotype divergence including multiple germin-like protein genes, at the wheat Sr2 adult plant stem rust resistance locus
Source: BMC Plant Biol. 2014 Dec 30;14:379. doi: 10.1186/s12870-014-0379-z (PMC4305260; doi:10.1186/s12870-014-0379-z)

## Slide 1
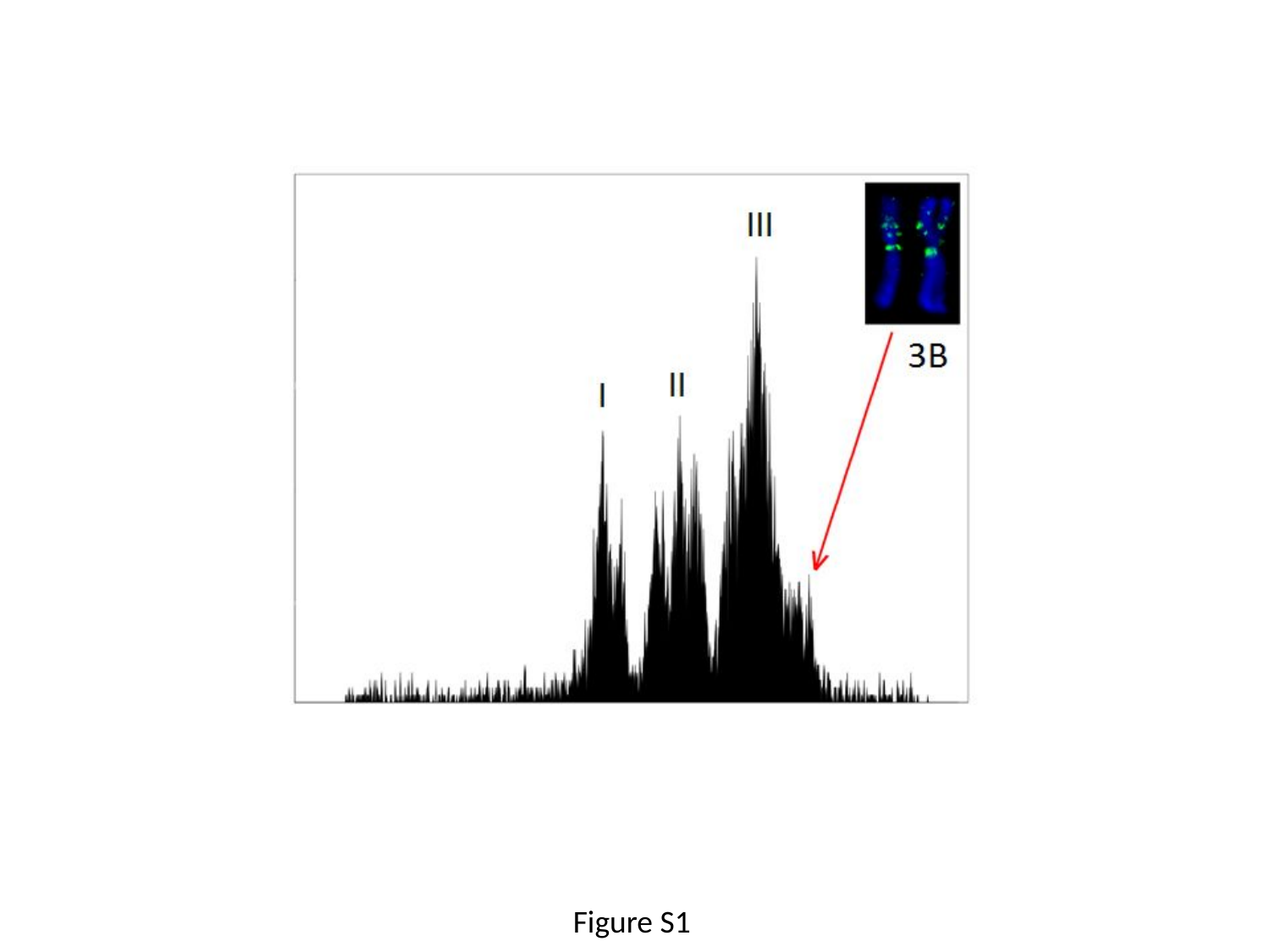

Figure S1

Supplement: Additional file 1: Figure S1. — Histogram of relative fluorescence (flow karyotype) from flow cytometric analysis of DAPI-stained mitotic chromosomes of Triticum aestivum cv. Hope. The flow karyotype consists of three composite peaks I – III representing groups of wheat chromosomes and peak of chromosome 3B. Inset: Images of flow-sorted 3B after FISH with a probe for GAA microsatellite (green). The chromosomes were counterstained by DAPI (blue). X axis: Relative fluorescence intensity. Y axis: Number of particles. [file 12870_2014_379_MOESM1_ESM.pptx]
