# Supplementary material for: Major haplotype divergence including multiple germin-like protein genes, at the wheat Sr2 adult plant stem rust resistance locus
Source: BMC Plant Biol. 2014 Dec 30;14:379. doi: 10.1186/s12870-014-0379-z (PMC4305260; doi:10.1186/s12870-014-0379-z)
Supplement: Additional file 2: Table S1. — Primer sequences used for amplification of markers that were genetically mapped. [file 12870_2014_379_MOESM2_ESM.docx]

**Additional file 2: Table S1** Primer sequences used for amplification of markers that were genetically mapped
